# Can severe asthmatic patients achieve asthma control? A systematic approach in patients with difficult to control asthma followed in a specialized clinic

**DOI:** 10.1186/s12890-016-0314-1

**Published:** 2016-11-16

**Authors:** Rodrigo Athanazio, Regina Carvalho-Pinto, Frederico Leon Arrabal Fernandes, Samia Rached, Klaus Rabe, Alberto Cukier, Rafael Stelmach

**Affiliations:** 1 Pulmonary Division - Heart Institute (InCor) do Hospital das Clinicas da Faculdade de Medicina da Universidade de São Paulo - São Paulo/BR, Av Dr Eneas de Carvalho Aguiar, 44 – 5° andar (Pneumologia), São Paulo, 05403-900 SP Brazil; 2 Lungen Clinic Grosshansdorf and Department of Medicine, Christian Albrechts University Kiel, Airway Research Center North (ARCN) in the German Center for Lung Research (DZL), Kiel, Germany

**Keywords:** Asthma, Control, Difficult to control, Health related quality of life, Refractory asthma

## Abstract

**Background:**

Despite advances in asthma treatment, severe asthma (SA) still results in high morbidity and use of health resources. Our hypothesis was that SA patients would achieve adequate control with a systematic protocol, including oral corticosteroids, budesonide/formoterol maintenance and reliever therapy and a multidisciplinary approach to improve adherence.

**Methods:**

Non-controlled (NC) SA patients were enrolled to receive 2 weeks of oral corticosteroids and 12 weeks of formoterol + budesonide. Assessments included asthma control questionnaire (ACQ), asthma control test (ACT), daily symptom diary, lung function and health-related quality of life (HRQoL) questionnaires.

**Results:**

Of 51 patients, 13 (25.5%) achieved control. NC patients had higher utilization of health resources and higher exacerbation rates. Both controlled (C) and NC patients had significantly reduced ACQ scores after oral corticosteroid treatment. After 12 weeks, C patients continued improving. NC patients did not have significant changes. A similar pattern was found regarding lung function, use of rescue medication, and days free of symptoms. After 2 weeks of oral corticosteroids, an increase occurred in those who achieved the ACQ cut off; however, 53.8% of C patients had an ACQ < 1.57 versus 21.1% of NC patients (*p* = 0.03). Both groups had low HRQoL at baseline with improvement after intervention.

**Conclusions:**

Despite rigorous, optimized follow-up treatment, 75% of SA patients did not achieve adequate symptom control and presented with impaired quality of life. Conversely, application of a low-cost, easy to implement systematic protocol can prevent up to 25% of SA patients from up-titrating to new and complex therapies, thus reducing costs and morbidity.

**Trial registration:**

Retrospectively registered at ClinicalTrial.gov on 22 February 2010 (NCT01089322).

## Summary at a glance

There is still debate if severe asthma (SA) patients may acquire symptoms control with available treatment. Our findings support that the application of a systematic, low-cost, easy to conduct protocol can prevent up to 25% of uncontrolled SA patients from up titrating their treatments to new and complex therapies.

## Background

The knowledge of the pathogenesis, pathophysiology and treatment of asthma has made great progress in recent decades. However, several surveys have shown that a significant part of patients do not achieve adequate control of the disease despite proper management according to guidelines [1, 2]. Factors such as poor access to medications, lack of medication adherence and environmental control, patient’s tendency to underestimate their symptoms, improper use of inhaler devices and presence of comorbidities have been linked to the inadequate symptom control [3].

These factors have a particularly significant impact in severe asthma (SA), in which this small proportion of patients is subject to high morbidity and disproportionate use of health resources [4, 5]. In this subgroup of patients, the need for a systematic evaluation in a specialized centre, including confirmation of the diagnosis of asthma, analysis of comorbidities, patient education, and supervised treatment have been suggested. Patients with asthma who remain uncontrolled despite this approach are considered to have asthma refractory to treatment [3]. Recently, however, a retrospective analysis by the British Thoracic Society Network concluded that a systematic approach is associated with better asthma control, gains in quality of life (QoL), and reduced health care costs [6].

Several validated tools are available to evaluate asthma control, such as diaries for symptoms, clinical questionnaires, and inflammatory/functional measurements [7, 8]. Each one has particular advantages, but little data regarding their applicability in SA has been published. Pharmacological trials and systematic protocol assessments use variations in symptom-based questionnaires, considered as minimally clinically significant values, to evaluate interventions. The performance of these instruments and the interpretation of results obtained with them have been questioned [9].

We have previously published the characteristics of a group of patients who did not reach full clinical control and maintained persistent airflow limitations, despite regular monitoring and treatment for at least 4 years - Brazilian Severe Asthma São Paulo (BRASASP) cohort [10]. Here, we report the results of a systematic approach to these patients. Our hypothesis was that most patients would achieve adequate control with a systematic protocol including oral corticosteroids, maintenance and reliever inhaled corticosteroids (IC) plus long-acting beta-agonists (LABA), and a multidisciplinary approach to improve adherence.

## Methods

### Setting and participants

This was a prospective study including a group of SA patients already followed in a specialised SA centre (BRASASP cohort) at least for 1 year. Patients were between 18 and 65 years old, had confirmed SA treated for at least 1 year, documented airway reversibility, presence of at least one asthma exacerbation in the previous year, were non-smokers or former smokers of ≤ 30 pack-years, and receiving high-dose IC plus LABA. If the patient was a smoker, asthma symptoms must have been present before the onset of smoking, and the patient could not smoke more than 10 cigarettes/day. The local Institutional Review Board approved this study (CAPPesq 757/05). All enrolled patients signed an informed consent. The project was retrospectively registered at ClinicalTrial.gov on 22 February 2010 (NCT 01089322).

### Design overview

**Fig. 1:**
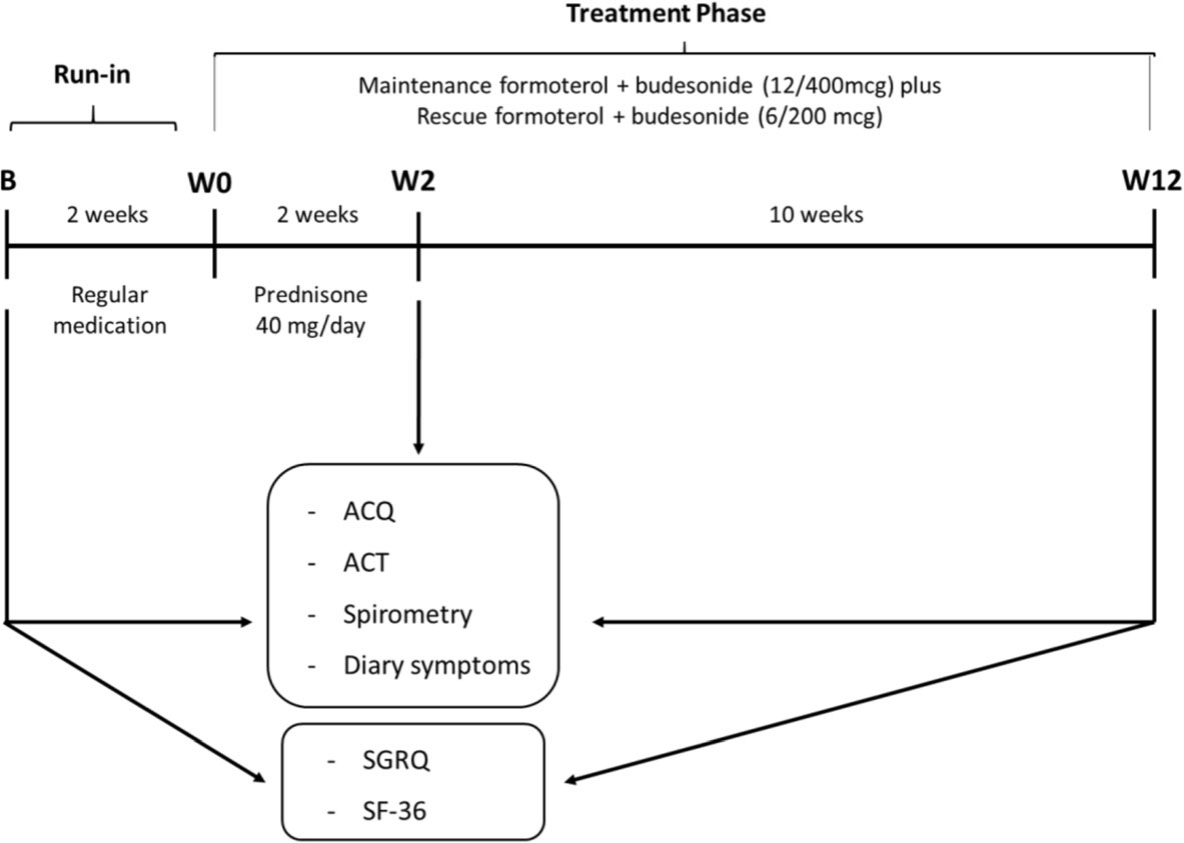
Study design. B: baseline, W: week, ACQ: asthma control questionnaire, ACT: asthma control test, SGRQ: St-George’s Respiratory questionnaire, SF-36: Medical Outcomes Study 36-Item Short Form Health Survey

The study design is shown in Fig. 1. The patients were enrolled between January 2007 and December 2009. They were selected to participate in the study if their asthma was not controlled according to GINA (Global Initiative for Asthma) criteria [11]. After a 2-week run-in period with regular medication to recheck control, non-controlled SA patients received maintenance therapy with formoterol plus budesonide 12/400mcg twice a day and reliever medication with formoterol plus budesonide 6/200mcg as needed for 12 weeks. They also received an oral corticosteroid (OC) (prednisone) (OC = 40 mg, day-1) in the first 2 weeks after run-in. Patients were evaluated according to ACQ, ACT, symptom diary, and spirometry, at baseline (B), after OC (Week [W] 2), and at the end of the study (W 12). At visit B and W 12, St George’s Respiratory Questionnaire (SGRQ) and Medical Outcomes Study 36-Item Short Form Health Survey (SF-36) were administered. In all visits they were seen by the same investigator, had the use of their inhaler checked, and compliance with medication assessed by a count of the remaining doses.

At baseline, all patients self-reported their past medical history and had their medical records evaluated. Comorbidities were evaluated prior to the protocol intervention as the standard clinical practice of our institution and aimed to confirm or exclude known diseases related to non-controlled asthma such as vocal cord dysfunction, gastroesophageal reflux disease, chronic rhinosinusitis, and other pulmonary diseases. Whenever present, these comorbidities were treated according to the same attending physician.

Exacerbation was characterized based on the expert panel of 2009 [12]. Patients who experienced exacerbation of asthma at any time during the study were treated with prednisone 40 mg/day for seven days, and the protocol evaluation measurements were postponed for 2 weeks. Antibiotics were prescribed according to physician judgement for each exacerbation. Total number of exacerbations was recorded.

### Outcome measures

We used ACQ 7 score to measure our primary objective of asthma control achievement at W12. Patients who maintained ACQ scores >1.57 were classified as the non-controlled group (NC) [13].

Secondary clinical outcomes included ACQ 7 score at W2, the Asthma Control Test (ACT) [14], lung function results, the St George’s Respiratory Questionnaire (SGRQ) [15], the Medical Outcomes Study 36-Item Short Form Health Survey (SF-36) [16], the ratio of days free of symptoms, and exacerbations.

### Statistical analysis

All continuous variables were evaluated for normality using Kolmogorov-Smirnoff or Shapiro-Wilk tests and are shown as mean ± standard deviation or median (IQ25% – 75%). ANOVA repeated measures were used to compare variables at three moments (B, W2, and W12) with Bonferroni’s correction. Unpaired t (independent groups) was used to analyse the measures between groups. Wilcoxon Signed-Rank and Mann–Whitney tests were used for non-parametric variable analysis. Categorical variables are presented as numbers and percentages and were analysed with the chi-square test. Correlations were performed using Pearson or Spearmen tests whenever appropriate. Multivariable logistic regression analysis was performed (C versus NC) with pre-defined relevant clinical variables (age, atopy, baseline ACQ, baseline FEV_1_ and asthma duration) to try do identify predictors of asthma control. The statistical package Sigma Stat version 3.5, Sigma Plot version 10, and PASW Statistics (SPSS) version 18 were used for statistical analysis. Statistical significance was considered *p* < 0.05.

## Results

Baseline characteristics of the whole group have been previously published [10]. From 74 enrolled patients, 54 (72.9%) completed the systematic treatment protocol. Patients were excluded during follow-up due to lack of adherence (6 patients), failure to fulfil eligibility criteria (5 patients), serious adverse events (4 severe exacerbations/1 ischemic heart disease/1 car accident/1 pregnancy), and two withdrew informed consent. From those who completed protocol, 51 (68.9%) patients had all data available to be analysed.

Of the 51 patients analysed during the 12-week systematic protocol, 38 (74.5%) did not reach the controlled criteria of our primary objective (ACQ < 1.57), thus were classified as the NC group, and 13 (25.5%) achieved control and were classified as the controlled group (C). Table 1 compares baseline characteristics of both groups. NC patients were younger, had shorter duration of disease and higher prevalence of atopy. NC had more women, although not statistically significant. Both groups mainly comprised overweight and obese patients. No statistical difference was found related to use of oral and inhaled corticosteroids as also as previous history of tobacco exposure.

**Table 1:** Baseline characteristics of non-controlled group (NC), controlled group (C) and not enrolled patients

n (%)	NC	C	Not enrolled (23 patients)
	38 (74.5)	13 (25.5)	
Age (years)^†^	42 ± 10	51 ± 10*	44 ± 10
Female, n (%)	28 (73)	6 (46)	21 (91)
Education (years)	7.6 ± 3.5	7.7 ± 3.9	8.3 ± 3.1
Asthma age of onset (years) ^§^	9.5 (1 – 31)	1.0 (1 – 18)	9.0 (1 – 22)
Asthma duration (years)^†^	28 ± 16	41 ± 15*	30 ± 13
BMI (kg/m^2^)^†^	30 ± 6	28 ± 6	30 ± 6
Atopy, n (%)	28 (73)	5 (38)*	9 (39)
Non/former smoker, n (%)	23 (60)/15(40)	11 (85)/2(15)	18 (78)/5 (22)
ICS (mcg/day) ^§^	1600 (1200 – 1600)	1600 (1000 – 1600)	1600 (1200 – 1600)
Oral steroids use, n (%)	7 (18)	4 (30)	7 (30)
Prednisone, mg/day^§^	20 (15 – 20)	15 (10 – 20)	20 (16 – 20)

*BMI* body mass index, *ICS* inhaled corticosteroid, *NC* non-controlled group, *C* controlled group

*p < 0.05 between NC and C groups; ^†^Mean ± SD; ^§^median (IQR)

NC patients had higher previous health care system utilization. Of NC patients, 71% had more than five hospitalizations compared with 38% in C patients (*p* = 0.05). Regarding hospitalization in the last year, no difference existed between groups (NC 37% versus C 23%). Nearly 40% of patients of both groups had undergone previous orotracheal intubation. Regarding comorbidities, there was no difference between self-reported diseases at baseline.

**Fig. 2:**
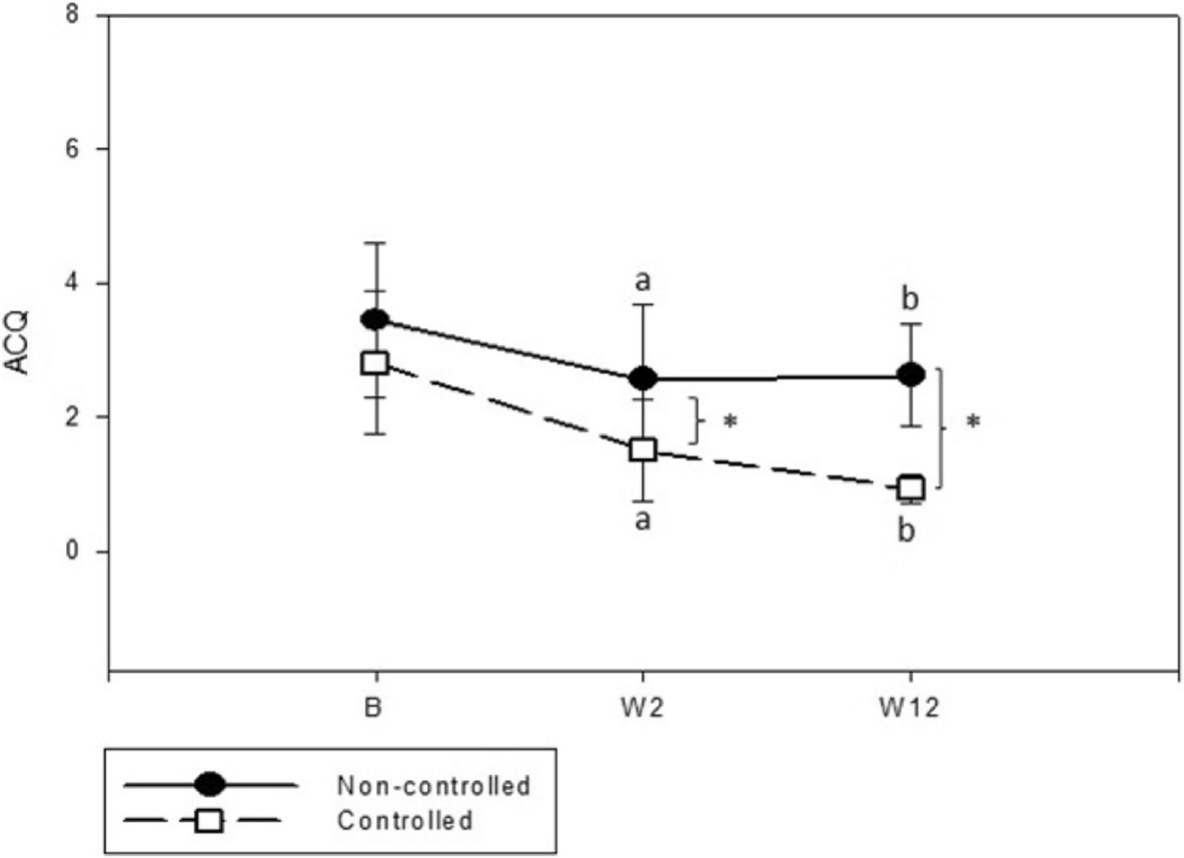
ACQ behaviour during systematic protocol between controlled and non-controlled groups. B: baseline, W: week. *p < 0.05 (Non-controlled vs Controlled); ^a^ *p* < 0.05 (W2 vs B); ^b^ *p* < 0.05 (W12 vs B)

ACQ analysis showed that both C and NC had a significant reduction in ACQ score after oral corticosteroid treatment (W2). After 12 weeks of follow-up, C continued to improve, while NC did not (Fig. 2). The ACQ score was significantly lower in C after treatment (W12) compared to NC (Table 2). At W2, an increase occurred in percentage of patients who achieved the cut off of asthma control in both groups; however, C had 53.8% of patients with ACQ <1.57 versus only 21.1% in NC group (*p* = 0.03). Despite not achieving asthma control, the majority of patients presented a clinically significant improvement in their symptoms at the end of the study, represented by a decrease of at least 0.5 in ACQ (C = 86.7% versus NC = 66.7% - *p* = 0.13). We were unable to identify predictors of asthma control in multivariable analysis.

**Table 2:** Comparison of ACQ and ACT scores, rescue medication use, days free of symptoms, and lung function parameters

Group (n)	NC (38)			C (13)		
	B	W2	W12	B	W2	W12
ACQ^†^	3.43 ± 1.14	2.54 ± 1.11^a^	2.62 ± 0.75 ^b^	2.8 ± 1.06	1.50 ± 0.76* ^a^	0.92 ± 0.22* ^b^
ACT^§^	10 (7 – 14)	13 (10 – 14)	13 (12 – 15)	13 (8 – 16)	17 (13 -– 20)* ^a^	21 (19 – 23)* ^b^
Rescue medication (puff/day) ^§^	3.1 (1.1 – 5.3)	2.5 (1.3 – 4.2) ^a^	2.2 (1.0 – 3.4) ^b^	2.7 (1.0 – 6.3)	1.0 (0.2 – 3.0) ^a^	0.4 (0 – 2.3)* ^b^
Days free of symptoms (%)^§^	0 (0 – 21)	7 (0 – 33)	0 (0 – 33)	0 (0 – 20)	42 (20 – 84)* ^a^	92 (22 – 100)* ^b^
FVC (%)^†^	66.8 ± 17.9	76.5 ± 21.3 ^a^	69.5 ± 20.4	68.2 ± 23.3	82.3 ± 15.9 ^a^	83.3 ± 18.5* ^b^
FEV_1_ (%)^†^	49.8 ± 17.3	65.4 ± 22.4^a^	55.2 ± 19.3^b^	54.8 ± 19.6	70.7 ± 18.6 ^a^	70.0 ± 21.8* ^b^
FEV_1_/FVC^†^	65 ± 13	65 ± 22	68 ± 13	60 ± 12	70 ± 18 ^a^	70 ± 11 ^b^

*FEV*_*1*_ forced expiratory volume in the first second, *FVC* forced vital capacity, *NC* non-controlled group, *C* controlled group, *B* baseline, *W* week

*p < 0.05 (NC vs C); ^a^ *p* < 0.05 (W2 vs B); ^b^ *p* < 0.05 (W12 vs B); ^†^Mean ± SD; ^§^median (IQR)

ACT values were significantly higher in C compared to NC after the course of oral corticosteroids (W2) and at the end of the protocol (W12) (Table 2). The percentage of patients with ACT score ≥20 was 30.8% and 69.2% in the C group at W2 and W12 compared to 13.2% in NC at both times (*p* <0.05). A significant correlation was noted between ACQ and ACT scores in NC patients at the end of the protocol (*r* = −0.587, *p* < 0.001), but not in C patients.

We observed a significant reduction in rescue medication use and an expressive increase of days free of symptoms in C, which remained until the end of the protocol. NC patients had no significant change in these variables. Comparing both groups during the protocol, C patients needed less rescue medication and had more days free of symptoms (Table 2).

**Fig. 3:**
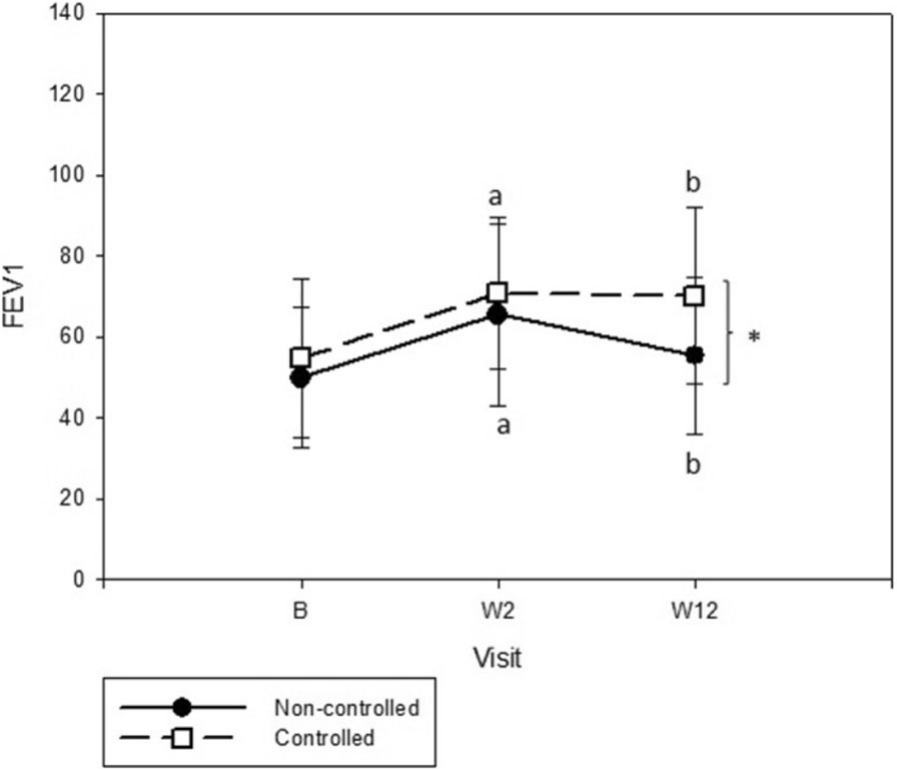
FEV_1_ behaviour during systematic protocol between controlled and non-controlled groups. B: baseline, W: week. *p < 0.05 (Non-controlled vs Controlled); ^a^ *p* < 0.05 (W2 vs B); ^b^ *p* < 0.05 (W12 vs B)

Evaluation of pulmonary function during follow-up showed significant improvement in FEV_1_ and FVC in both groups after oral corticosteroids. Remarkably, FEV_1_ and FVC were statistically higher in the C compared with NC at the end (Table 2). In C, FEV1 was stable between W2 and W12, although NC patients tended to lose lung function after oral corticosteroids (Fig. 3).

During the protocol measurements, 29 (56.9%) patients experienced at least one exacerbation. No difference existed between C and NC groups (60.5% versus 46.2%g. Nevertheless, there was a higher but not significant mean number of exacerbations/patient (1.03 ± 1.1) in NC compared with C (0.46 ± 0.5).

Both groups had low HRQoL at baseline involving all SGRG domains. At the end of the intervention, the C had significantly better HRQoL in all SGRQ domains (Table 3). The C group tended to have higher percentage of patients with a ≥4-point change in total control on the SGRQ (89% vs 61%, *p* =0.058) compared with NC, indicating clinical improvement in HRQL. In the SF-36 questionnaire, the two groups reported lower HRQoL at baseline. As observed in the SGRQ questionnaire, C had greater improvement in SF-36 measurements than NC had at the end of protocol.

**Table 3:** Health-related quality of life (HRQoL) scores between NC and C and asthmatic patients

	NC		C	
	B	W12	B	W12
SGRQ – Total	68 ± 13	62 ± 14	60 ± 13	38 ± 15*
Symptoms	65 ± 18	58 ± 19	58 ± 21	20 ± 17*
Activity	82 ± 17	76 ± 19	73 ± 19	56 ± 19*
Impact	66 ± 12	55 ± 16	57 ± 15	33 ± 16*
SF-36				
Physical health				
Functional capacity	31 ± 22	38 ± 22	46 ± 20*	58 ± 19*
Physical aspects	21 ± 29	43 ± 40	26 ± 31	68 ± 37
Pain	41 ± 22	47 ± 25	50 ± 23	43 ± 30
General health status	38 ± 18	45 ± 19	49 ± 22	57 ± 19
Mental health				
Vitality	42 ± 20	46 ± 20	46 ± 17	60 ± 16*
Social aspects	51 ± 27	60 ± 26	61 ± 26	79 ± 18*
Emotional aspects	36 ± 44	49 ± 41	35 ± 39	83 ± 33*
Mental health	52 ± 24	56 ± 23	54 ± 22	68 ± 16

*SGR*Q St George’s Respiratory Questionnaire, *NC* non-controlled group, *C* controlled group, *B* baseline, *W* week

Data expressed as mean ± SD; *p < 0.05 (NC vs C)

The ACQ score showed a good correlation with HRQoL baseline and ending questionnaires, mainly with SGRQ. Total SGRQ score was positively correlated to ACQ (*r* = 0.681; *p* <0001) and all independent domains (symptoms: *r* = 0.724; activity: *r* = 0.438; impact: *r* = 0.666). SF-36 correlated with ACQ score in all domains, except pain.

## Discussion

In our SA cohort, despite regular real-life treatment in our specialized clinic, one-fourth of patients achieved clinical control in accordance with current asthma control scores with systematic follow-up. Additionally, we observed an improvement in QoL scores and lung function in both groups, although the C group had a more prominent and sustained behaviour. Our findings support a controversial discussion about the real possibility of achieving asthma control in the majority of patients with severe disease and/or whether we are using the right tools to evaluate it.

Patients included in this study had uncontrolled asthma despite regular care in our reference center at a university hospital. We designed the study with the expectation that following a systematic protocol and personalized care (patients were followed at all visits by the same researcher) would lead to disease control.

All patients were uncontrolled despite regular use of inhaled corticosteroids associated with LABA, including the run-in period. During the 12-week study, we applied a maintenance and reliever strategy with formoterol and budesonide proven to be effective in patients with moderate to severe asthma, especially in symptom scores and number of exacerbations [17]. In the first 2 weeks, we administered oral corticosteroids to optimize the response [11, 18, 19].

This strategy was clearly effective in a subset of patients. The ACQ incorporates seven items (five symptom questions, one reliever use question, and pre-bronchodilator FEV_1_ measurement) and a 1-week recall time. It assesses the adequacy of asthma control and the change in asthma control over time. A change in score of > 0.5 is considered to be clinically important and an ACQ score of 1.57 discriminates between controlled and non-controlled asthma [13]. Thirteen out of 51 patients achieved a mean ACQ value less than 1.57 after 2 weeks of oral corticosteroids, and showed a 1.6 difference in the score from baseline at 12 weeks. This response was accompanied by obvious improvements in other outcomes, such as ACT (scores from 20 to 25 were classified as well-controlled asthma; 16–20 as not well controlled; and 5–15 as very poorly controlled asthma; the minimum clinically significant difference is 3 points) [14], number of days free of symptoms and use of rescue medication. The improvement in these outcomes was reflected in the scores of quality of life questionnaires. Spirometric parameters followed this improvement, but the average FEV1 reached only 70% of predicted values. These results are in agreement with previous studies that demonstrated the benefit of applying a systematic protocol for patients with difficult to control asthma referred to specialized centers [20, 21]. Our data show that even in specialized clinics taking care of patients in real-life conditions a personalized and systematic strategy is effective, leading to control of patients who would otherwise be considered non-controlled and suitable for a treatment increase.

In the NC group the mean ACQ was 2.54 after the use of oral corticosteroids and 2.62 at 12 weeks. However we observed a clinically significant improvement in the levels of ACQ (0.9 points) and ACT (3 points) after the use of oral corticosteroids, as well as significant improvement in FEV1. On the other hand, virtually no patients were daily free of symptoms during the period. In the following 10 weeks, despite a higher dose of inhaled corticosteroids compared to the control group (shown by the number of rescue doses of formoterol/budesonide), there was a trend to a clinical and spirometric worsening. Similar response to a course of systemic corticosteroids have been previously reported. ten Brinke et al. demonstrated a reduction in rescue medication and an increase in FEV1 2 weeks after intramuscular triamcinolone in patients with severe asthma who were using inhaled corticosteroids or chronic oral prednisone [22]. In a recently published study, the researchers of BIOAIR found a higher than 12% increase in FEV1 after a 2 week course of oral corticosteroids in 15 of 84 patients with severe asthma [18]. The magnitude of spirometric response in our group of uncontrolled patients after 2 weeks of systemic corticosteroids demonstrates that a significant functional response to a course of oral corticosteroids is not necessarily reflected in adequate control of the disease in the medium and long term.

We identified a similar profile of patients characterized by predominantly female gender, long term disease, and overweight and atopic patients compared to other large cohorts of SA such as SARP and ENFUMOSA [23, 24]. We found that comorbidities are highly prevalent in patients with severe asthma not well controlled. Otherwise, this prevalence was not affected by our intervention, suggesting that the comorbidities that we evaluated may not interfere directly in asthma control. These data are in agreement with previous studies, which showed that in poorly controlled asthmatics there is a high prevalence of comorbidity, but no difference in prevalence between patients who respond or not to treatment [20].

There are some limitations in our study. The relatively low number of patients in the C group limits the statistical power to some correlations, although the data were consistent with those previously published [2, 18, 25]. Since this is a single centre study, characterized by a long-term SA population and public drug access, this might limit applicability to others health care settings. We have not covered the full spectrum of comorbid conditions, nor included an asthma education program at the visits, which could increase the percentage of controlled patients. In fact, we have demonstrated that weight reduction leads to asthma control regardless of asthma treatment [26]. These limitations did not interfere with the main message of our study, namely that a personalized approach allows asthma control in a significant proportion of patients with no change in the medication for asthma. We were not able to identify which tool is the best to follow up patients with SA among spirometry, symptom diary, HRQol, ACQ and ACT. Nevertheless, we believe that is not possible that only one tool would be able to discriminate all aspects of such a heterogeneous disease and that the management should be based on a combination of them. Finally, it is a not blinded study. However, since the patients were already following standard treatment guidelines in a reference center and were not controlled, we consider it appropriate not to include a control group.

Our study has important clinical implications. Patients classified as GINA steps 4 and 5 followed at our asthma clinic for at least 4 years, with free access to anti-inflammatory therapies, educational programs and proper evaluation of factors associated with non-control composed our population [27–31]. Given the lack of control add on therapy would be the next natural step to be implemented in everyday practice. Our systematic low cost and widely available protocol with optimised IC + LABA + short course oral corticosteroid was able to control 25% of patients. The rising cost of medicine and the pressure to incorporate new technologies are crucial issues when discussing non-infectious chronic diseases [32]. Even in developed countries there is a consensus that the incorporation of high-cost asthma treatments is justified only to a select group of patients [33, 34]. Our results highlight the importance of a thorough assessment before considering the indication of these costly treatments.

## Conclusions

Our findings support that in patients with severe uncontrolled asthma in daily practice an automatic step up in treatment may lead to unnecessary increase in expenses and risks related to new treatments, since the control can be achieved with a clinical targeted treatment approach. Nevertheless, despite rigorous and optimized follow-up treatment, the majority of patients with SA did not achieve adequate symptom control, and had high exacerbation rates and impaired QoL. These findings indicate the necessity to redefine the goals and monitoring tools related to SA. The application of a systematic, low-cost, easy to conduct protocol can prevent up to 25% of SA patients from up titrating their treatments to new and complex therapies, thus reducing costs (possibly) and morbidity (certainly).

## Abbreviations

ACQ: Asthma control questionnaire
ACT: Asthma control test
B: Baseline
BRASASP cohort: Brazilian Severe Asthma São Paulo cohort
C: Controlled
FEV1: Forced expiratory volume in the first second
FVC: Forced vital capacity
GINA: Global initiative for asthma
HRQol: Health related quality of life
IC: Inhaled corticosteroids
IQ: Interquartile
LABA: Long, acting beta, agonists
NC: Non, controlled
OC: Oral corticosteroids
Qol: Quality of life
SA: Severe asthma
SF-36: Medical outcomes study 36-item
SGRQ: St George’s respiratory questionnaire
W: Week
